# Systematic Analysis of Fly Models with Multiple Drivers Reveals Different Effects of Ataxin-1 and Huntingtin in Neuron Subtype-Specific Expression

**DOI:** 10.1371/journal.pone.0116567

**Published:** 2014-12-31

**Authors:** Risa Shiraishi, Takuya Tamura, Masaki Sone, Hitoshi Okazawa

**Affiliations:** 1 Department of Neuropathology, Medical Research Institute and Center for Brain Integrative Research, Tokyo Medical and Dental University, Yushima, Bunkyo-ku, Tokyo, Japan; 2 Department of Biomolecular Science, Faculty of Science, Toho University, Miyama, Funabashi, Chiba, Japan; Children's Hospital of Pittsburgh, University of Pittsburgh Medical Center, United States of America

## Abstract

The fruit fly, *Drosophila melanogaster*, is a commonly used model organism for neurodegenerative diseases. Its major advantages include a short lifespan and its susceptibility to manipulation using sophisticated genetic techniques. Here, we report the systematic comparison of fly models of two polyglutamine (polyQ) diseases. We induced expression of the normal and mutant forms of full-length Ataxin-1 and Huntingtin exon 1 in cholinergic, dopaminergic, and motor neurons, and glial cells using cell type-specific drivers. We systematically analyzed their effects based on multiple phenotypes: eclosion rate, lifespan, motor performance, and circadian rhythms of spontaneous activity. This systematic assay system enabled us to quantitatively evaluate and compare the functional disabilities of different genotypes. The results suggest different effects of Ataxin-1 and Huntingtin on specific types of neural cells during development and in adulthood. In addition, we confirmed the therapeutic effects of LiCl and butyrate using representative models. These results support the usefulness of this assay system for screening candidate chemical compounds that modify the pathologies of polyQ diseases.

## Introduction

Model animals are indispensable tools for studying human neurodegenerative diseases, and the selection of an appropriate model animal is critical. The fruit fly, *Drosophila melanogaster*, is a widely used model animal for neurodegenerative diseases. Mammals and insects share many fundamental genetic and cellular mechanisms. Approximately 75% of the genes that cause human diseases have orthologs in the *Drosophila* genome [1]. Due to its short lifespan and the low cost of *Drosophila* culture, it is an efficient and reasonable animal model for genetic studies. It is also possible to discriminate between developmental and late-onset progressive effects by changing the genetic driver or using a temperature sensitive system.

Drosophila models have been used to study various types of neurodegenerative diseases, such as the polyQ diseases, Alzheimer's disease, and Parkinson's disease [2]–[5]. Most of these studies employed the rough eye phenotype to evaluate the genetic effect because this phenotype does not affect fly viability, and only a single generation is necessary to test stock collections [6]–[13]. In fact, rough eye models have been used to identify many modifier genes [12]–[14].

In addition to the rough eye models, other behavioral phenotypes have been developed for the study of neurodegeneration. In some models, mutant polyQ proteins were expressed in distinct neurons and analyzed quantitatively through phenotypes such as eclosion, longevity, motor activity, circadian rhythm, and mating behavior [15]–[17]. The quantitative nature of these phenotypes offers distinct advantages for screening candidate therapeutic genes or chemicals. However, the phenotypes induced by different drivers have not been systematically compared or summarized. Comparisons of different disease models are very complicated because degradation rate of each pathogenic protein can differ between diseases [18]–[19]. Nevertheless, such cross-model comparisons are important for understanding the specificity and commonality of diseases.

In this study, we systematically analyzed Drosophila neurodegeneration models and summarized their efficiencies for future application in genetic or chemical screening *in vivo*. Huntingtin exon 1 (Htt) and full-length Ataxin-1 (ATXN1) are causative genes of Huntington's disease (HD) and spinocerebellar ataxia type 1 (SCA1), respectively. We tested the expression of their polyQ-elongated forms under the control of various neuron-specific Gal4 drivers.

## Materials and Methods

### Fly stocks

All flies were raised on a cornmeal medium (9.2% cornmeal, 3.85% yeast, 3.8% sucrose, 1.05% potassium tartrate, 0.09% calcium chloride, 7.6% glucose, 2.416% nipagin, and 1% agar). All fly stocks were maintained at 25°C and 60%±10% humidity under a 12∶12 hr light-dark cycle, unless otherwise noted. The 1407-Gal4 (#8751), D42-Gal4 (#9263), cha-Gal4 (#6798), ple-Gal4 (#8848) and UAS-EGFP (#5431) strains were obtained from the Bloomington Drosophila Stock Center (Indiana University, IN, USA). We also acquired the elav-Gal4 (#8765, P{GAL4-elav.L}2) [20], OK6-Gal4, repo-Gal4, UAS-Htt-20Q (exon 1) (1 line), and UAS-Htt-103Q (exon 1) fly strains [21], [22]. Transgenic UAS-ATXN1-82Q (full length) flies (*y^1^w^1118^UAS::hATXN1-82Q full length*) were also described previously [8]. We generated UAS-ATXN1-33Q transgenic flies using the same method described previously [23]. hATXN1-33Q-myc (full length) was introduced into the docking site VK00005 at 75A1059 using the PhiC31 integrase-mediated transgenesis system [24]. The Cantonised *w^1118^* strain *w(CS10)* [25], also known as 2202u, was used for the negative controls because all stocks used in this study were backcrossed with 2202u more than 6 times.

### Fly eclosion rate

Twenty 3^rd^ instar wall-climbing larvae were carefully gathered in a food vial. All gathered larvae pupated within a few hours. We counted the number of eclosed adult flies for 1 week.

### Longevity test

Lifespan was measured as previously described [26]. Approximately 20 flies were reared in one food vial. The food vial was changed every 2 or 3 days, and the number of dead flies was counted.

### Viability test

The OK6, elav, 1407, and cha-Gal4 strains were balanced with a 2^nd^ linked marker: gla, Bc33. D42 and repo-Gal4 were balanced with a 3^rd^ linked marker: TM3, sb. Finally, we crossed UAS-ATXN1-82Q or UAS-Htt-103Q virgin females with the balanced driver males. The driver/marker (gla or sb) ratio of the F1 flies was counted. Because each driver or marker has its own viability (S3A Fig.), we used the ratios from the control experiments to normalize the results. The normalization was performed by dividing the ratio of each model by the ratio of the corresponding control. Thus, the non-toxic level in the final figure is shown as 1.0 on the Y-axis.

### Pharmacological treatment

For pharmacological treatment, the flies were raised on medium containing the selected drug. The 10× drug stock solutions were diluted to their final concentrations with normal medium (1∶9 drug solution: medium). Milli-Q water was used as the solvent. For the viability assay, the drug-containing food was used for egg laying. For the longevity assay, the drug-containing food was used to maintain the flies. LiCl and butyrate were obtained from Wako (Japan). SP600125 was obtained from Selleck Chemicals (USA).

### Startle-induced negative geotactic response

A single fly was transferred into a test column (150 mm in length and 25 mm in diameter) lined with nylon mesh. We then tapped the column to drop the fly to the bottom of the column. The time the fly took to reach a height of 5 cm from the bottom was recorded using a stopwatch. When a fly did not reach 5 cm within 15 sec, the time was recorded as 15 sec. To estimate the potential motor performance, the test was repeated 3 times for each fly and the shortest record was utilized. Seven to 20 flies were maintained in a food vial until the testing commenced. The number of flies tested is listed in S1 Table.

### Spontaneous activity test

Virgin males were placed individually in glass tubes. One end was filled with medium, and the other end was filled with cotton. The motion of the flies was detected and counted by infrared light beam breaks every 30 min using a Drosophila Activity Monitoring System (Trikinetics, Waltham, MA). The flies were kept under a 12-hour light/12-hour dark cycle (LD) for 7 days. The peak activity was identified as the activity during the time periods, 7:00–9:00 and 19:00–21:00.

### Real time PCR

Total RNA was prepared from 10 whole flies (3 days old) using an RNeasy mini Kit (Qiagen, Germany). To eliminate genomic DNA contamination, each sample was subjected to on-column DNA digestion with DNase I (Qiagen). First-strand cDNA was synthesized from 200 ng of total RNA using Superscript II VILO (Invitrogen, CA, USA). Quantitative PCR analyses were performed for the full-length ATXN1 and Htt exon 1 using the 7500 Real-Time PCR System (Applied Biosystems, CA, USA) and Thunderbird SYBER Green (TOYOBO, Japan). The primers were designed from vector sequence in order to quantify Htt and ATXN1 expression using same primer set. The sequences were forward primer 5′-AACTGATGAATGGGAGCAGTG-3′ and reverse primer 5′-GGAAAGTCCTTGGGGTCTTC-3′. For the absolute quantification, pUAST -Htt103Q exon 1 was used to prepare the standard curves. The cDNA amounts were normalized based on qPCR using forward primer 5′-CCGAGCGCGGTTACTCTTT-3′ and reverse primer 5′-CAAATAGCACAGCTTCTCCTTGAT-3′.

### Morphology

The brain and ventral ganglion (VG) were dissected from adult flies in PBS. The central nervous system (CNS) and ventral nerve cord (VNC) were dissected from the 2nd or 3rd instar larvae in PBS. The dissected materials were then mounted in VectaShield Mounting Medium (Vector Laboratories). Three-dimensional images were captured with a Zeiss LSM 510 confocal microscope (Carl Zeiss) using a 20× C-Apochromat dry objective lens [numerical aperture (NA), 1.2]. The legs were directly examined under a fluorescence stereomicroscope (SZX10, Olympus).

### Statistics

To identify significant differences between the controls and the models, we compared each model with 3 different controls. First, we tested the difference between the UAS control and the model. Because the UAS control was a common control for all (HD or SCA1) models, we employed Dunnett's multiple comparison. Next, we compared each model with the corresponding driver and the short Q controls. Because each driver and short Q are independent, we simply employed a two-tailed Welch's *t*-test for comparison. For comparing the lifespans, we employed the non-parametrical Steel's test and the Wilcoxon rank-sum test instead of Dunnett's multiple comparison or Welch's *t*-test. When the value of a model was significantly different from the UAS, the driver, and the short Q controls, we annotated the data with an asterisk(s). To evaluate pharmacological effects on lifespan, we employed the non-parametrical Steel's test. For chemical screens using viability assays, we employed Dunnett's test.

## Results

### Systematic generation of multiple fly models

We previously reported novel polyQ fly disease models expressing Htt-103Q (exon 1) or full-length ATXN1-82Q driven by specific Gal4 drivers and found some differences in their phenotypes [21], [22]. These findings prompted us to systematically screen the effects of Htt-103Q or ATXN1-82Q in different types of neural cells using the Gal4/UAS system. For this purpose, we employed seven different Gal4 drivers in this study. Elav-Gal4 is one of the most commonly used pan-neuronal drivers for driving gene expression throughout the nervous system. Elav–Gal4 expression is usually restricted to post-mitotic neurons, but it is also transiently expressed in embryonic glial cells [27]. 1407-Gal4 is an enhancer trap strain that drives pan-neuronal expression. In addition to mature neurons, it is also expressed in neuroblasts [28], [29]. OK6-Gal4 and D42-Gal4 are motor neuron-specific drivers. OK6 drives highly specific expression in motor neurons, while D42 also drives expression in sensory neurons [30]. Cha-Gal4, which is under the control of the choline acetyltransferase promoter, is generally considered a cholinergic driver. Cha-Gal4 expression is found from embryonic stages to adulthood in the central nervous system (CNS), VNC (Ventral Nerve Cord), and peripheral sensory neurons [31]. Ple-Gal4, which is also known as TH-Gal4, is a dopaminergic neuron-specific driver that is predominately expressed in tyrosine hydroxylase (TH)-positive neurons. The expression of ple-Gal4 shows clear and specific patterns in the adult and larval CNS and VNC [32]–[34]. Repo-Gal4 is a well-established glial driver that is expressed in all glial cells except the midline glia [35], [36]. Repo-Gal4 expression is under the control of Reversed Polarity (repo), a homeobox gene required for the differentiation and maintenance of glial function. The expression of repo-Gal4 is maintained in glioblasts and immature to mature glial cells but not in neuroglioblasts [21].

We systematically generated fourteen polyQ fly models by crossing transgenic flies bearing one of each of the seven different Gal4 drivers with transgenic flies carrying a normal or mutant form of one of the polyQ disease genes (Htt and ATXN1) downstream of a UAS promoter. Then we analyzed the fourteen models with four different quantitative tests to evaluate eclosion, longevity, geotaxis, and spontaneous activity (Table 1).

**Table 1:** Summary of phenotypes.

		developmental		progressive							
		eclosion		longevity		geotaxis		spontaneous activity			
								total activity		peak ratio	
		Htt	ATXN1	Htt	ATXN1	Htt	ATXN1	Htt	ATXN1	Htt	ATXN1
pan-neuronal	elav	−	+	±	−	−	−	−	−	−	−
	1407	−	+++	±	+	−	−	−	−	+	+
motor neuron	OK6	−	+	++	++	+	+	−	−	−	−
	D42	+	+	++	+	−	+	−	−	−	−
cholinergic	cha	+++	−	+++	−	++	±	−	−	+	+
dopaminergic	ple	+++	lethal	++		++		−		+	
glia	repo	−	lethal*	+++		+++		−		−**	

− not significant in comparison with three controls (UAS, GAL4 and short Q).

± significant but slight (<20%) difference.

+ less than 2 fold difference.

++ more than 2 fold difference.

+++ more than 3 fold difference.

* severe toxicity in short Q control.

** shift of peak time in short Q control.

### Gene expression patterns and levels in multiple fly models

To confirm the pattern and level of expression of the seven drivers (elav-Gal4, 1407-Gal4, OK6-Gal4, D42-Gal4, cha-Gal4, ple-Gal4 and repo-Gal4), we crossed each driver line with UAS-EGFP flies. We then dissected nervous tissue samples from the adult flies and directly observed EGFP fluorescence by confocal microscopy using 3D images (S1 Fig.). The expression patterns were mostly consistent with those expected. However, the expression levels among the drivers (S1 Fig.). Therefore, we performed quantitative PCR of the polyQ gene in each model and short Q control flies in order to confirm the expression level varied of the different drivers. The qPCR results again revealed divergent expression levels among the drivers and the genes (S2 Fig.). However, there were clear gene-specific and tissue-specific effects because Htt-103Q and ATXN1-82Q expression levels were similar when the same drivers were used (Table 2).

**Table 2:** Summary of the relationship between expression level and phenotype.

		expression level	phenotype	
			developmental	progressive
elav	Htt	+	−	+
	ATXN1	+	+	−
1407	Htt	++	−	±
	ATXN1	+++	+++	+
OK6	Htt	++	−	++
	ATXN1	++	+	++
D42	Htt	++	+	++
	ATXN1	++	+	+
cha	Htt	++	+++	+++
	ATXN1	++	−	+
ple	Htt	+++	+++	++
	ATXN1	lethal	lethal	lethal

expression level:

+; 2000–2500 copies/µg total RNA.

++; 4000–6500 copies/µg total RNA.

+++; 8000– copies/µg total RNA.

phenotype:

development = eclosion.

progressive = The strongest of progressive phenotypes (longevity, geotaxis or spontaneous activity).

These data are employed from table 1.

− not significant.

± significant but slight (<20%) difference.

+ less than 2 fold difference.

++ more than 2 fold difference.

+++ more than 3 fold difference.

### Eclosion rate of the fly models

**Figure 1:**
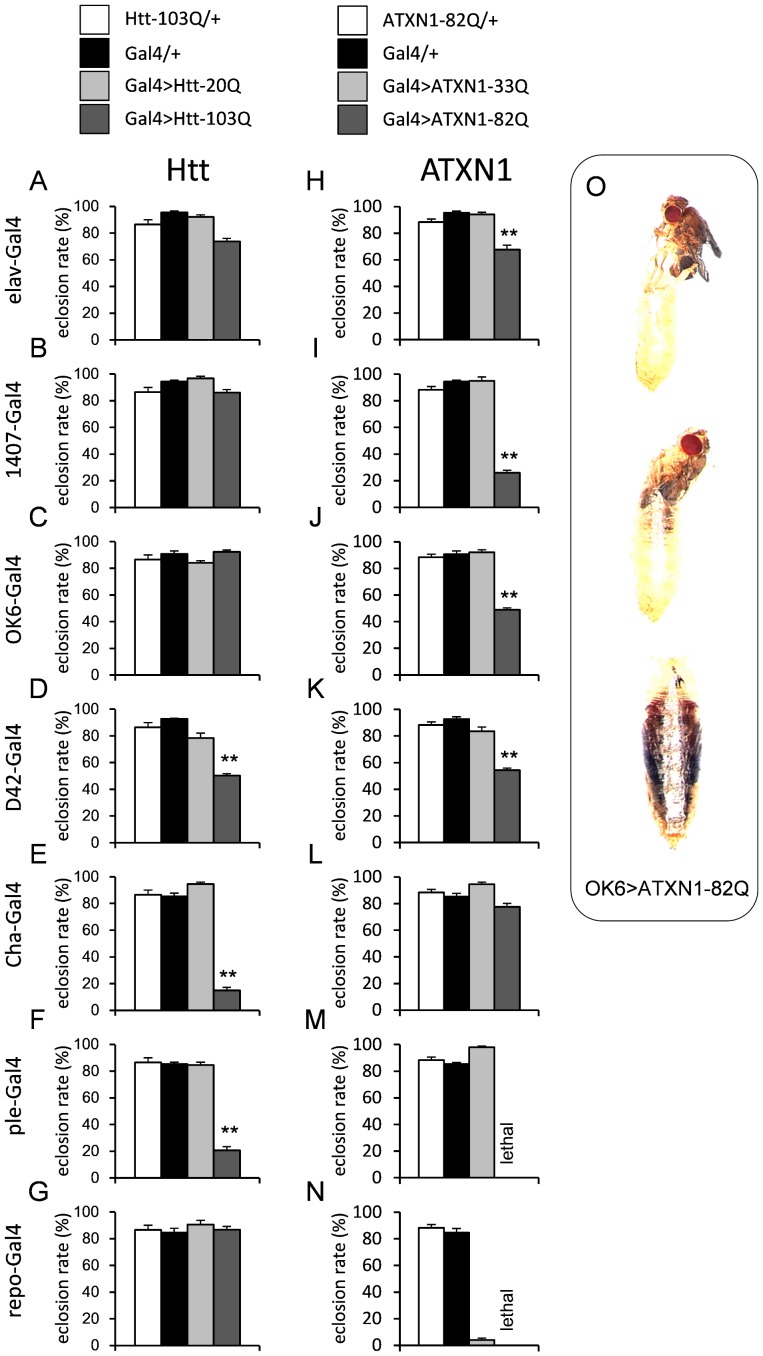
The eclosion rates of each model and the genetic controls are shown (A–N). The Y-axis represents the probability of eclosion. The drivers are indicated on the left side of each graph. Each bar represents the mean ± SE. Double asterisks indicate significant reduction in comparison to all three controls by Dunnett's test or Welch's t-test (p<0.01). The numbers of vials tested are listed in S1 Table. The results of each statistical analysis are summarized in S2 Table. Examples of the eclosion failure of OK6>ATXN1-82Q are shown (O).

We found that some pupa failed to eclose in some models (Fig. 1). D42>Htt103Q, cha>Htt-103Q, ple>Htt-103Q, elav>ATXN1-82Q, 1407>ATXN1-82Q, OK6>ATXN1-82Q, and D42>ATXN1-82Q produced significantly reduced eclosion rates (Fig. 1D, E, F, H, I, J and K, S1 and S2 Tables). Moreover, the ple-Gal4-driven expression of ATXN1-82Q induced larval death at the second instar stage (Fig. 1M). Glial expression of Htt-103Q driven by repo-Gal4 did not cause an eclosion defect (Fig. 1G), but glial expression of ATXN1-82Q caused complete lethality (Fig. 1N). Other combinations did not significantly affect eclosion (Fig. 1A, B, C, G and L).

In general, the eclosion defect was more prominent in the SCA1 models, although cha-Gal4 had a stronger effect in the HD models (Fig. 1E, L). Surprisingly, the glial expression of ATXN1-33Q induced a severe eclosion defect (Fig. 1N). This result clearly suggests that the toxicity of normal ATXN1 in glial cells is very high.

### Lifespan of the fly models

**Figure 2:**
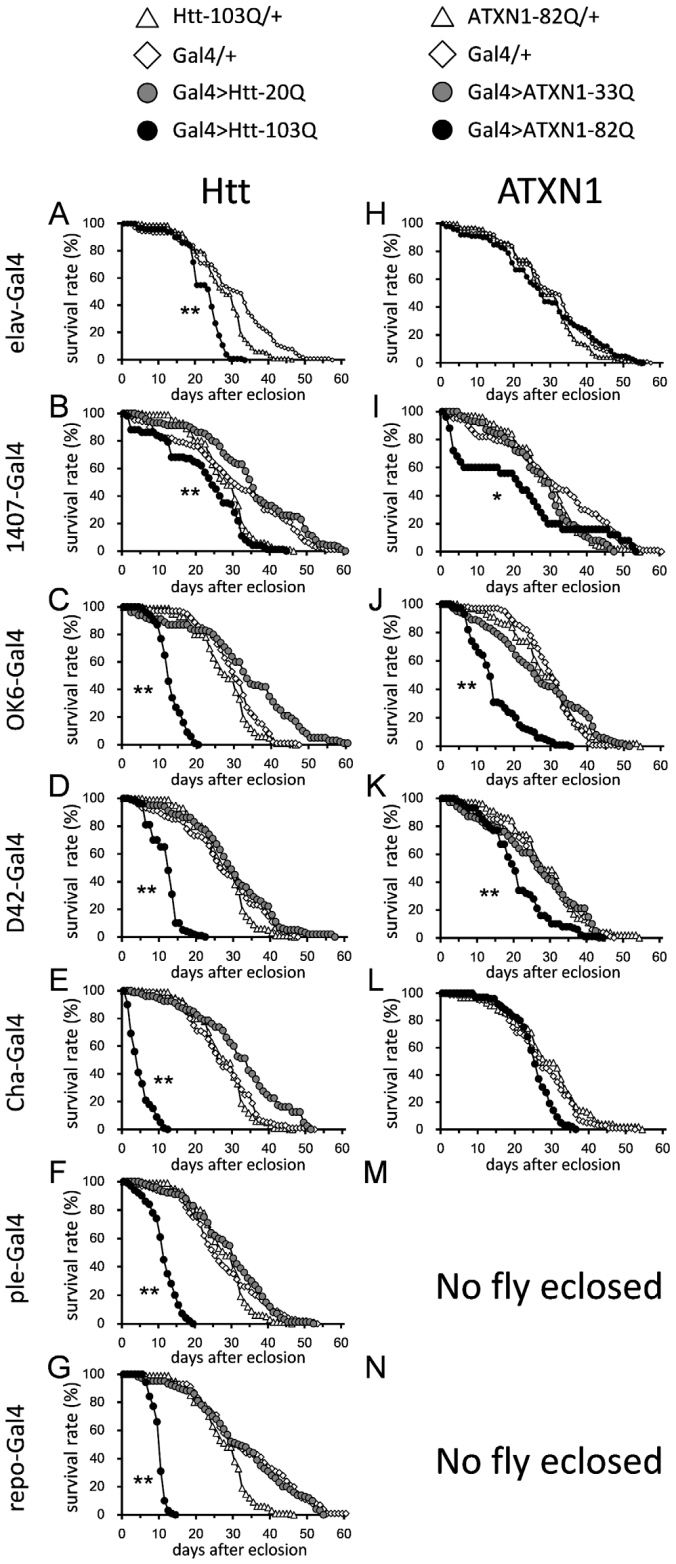
The longevities of each model and the genetic controls are indicated. The X-axis is the number of days, and the Y-axis is the survival percentage. The UAS-Htt-103Q or 20Q flies crossed with the drivers are shown in A–G, and the UAS-ATXN1-82Q or 33Q flies crossed with the drivers are shown in H–N. The name of the driver used is indicated on the side of each graph. The numbers of flies tested are listed in S1 Table. The results of the statistical analyses are listed in S3 Table.

Next, we analyzed the lifespan of each of the models. Elav-Gal4-driven expression shortened the lifespan of the HD models (Fig. 2A, S1 and S3 Tables) but not that of the SCA1 models (Fig. 2H, S1 and S3 Tables). 1407-Gal4-driven expression shortened the lifespan of both the HD and SCA1 models (Fig. 2B, I, S1 and S3 Tables). The difference between elav-Gal4 and 1407-Gal4 drivers might be related to the fact that elav expression is limited to mature neurons, while 1407 is also expressed in neuroblasts. There are also other variations in their expression patterns that could account for the different phenotypes. The expression of mutant polyQ proteins in motor neurons, driven by either OK6 or D42-Gal4, significantly shortened the lifespan of both the HD and SCA1 models (Fig. 2C, D, J and K, S1 and S3 Tables). In contrast to the more severe eclosion phenotypes seen in the SCA1 models, lifespan shortening was more severe in the HD models. This suggests that the continuous expression of Htt-103Q in adulthood is more toxic than the continuous expression of ATXN1-82Q. Cha-Gal4 resulted in an extremely short lifespan in the HD model (Fig. 2E, S1 and S3 Tables), whereas no phenotype was observed in the SCA1 model (Fig. 2L). This result again suggests that cholinergic neurons are resistant to mutant ATXN1. Ple-Gal4 and repo-Gal4 caused significantly shortened lifespan of the HD models (Fig. 2F, G, S1 and S3 Tables) and lethality in the SCA1 models (Fig. 1M, N, S1 and S2 Tables).

### Motor activity of the fly models

**Figure 3:**
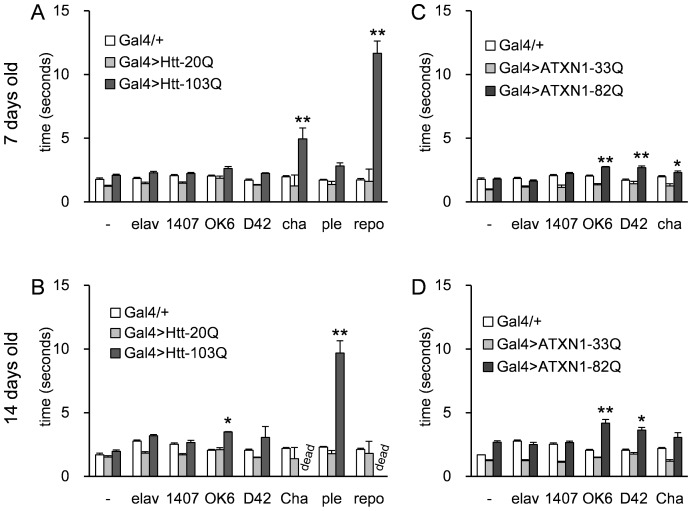
The motor performance was estimated using a negative geotactic assay. The HD models (A, B) and the SCA1 models (C, D) were tested at 7 days (A, C) and 14 days (B, D). Each Gal4 driver tested is listed along the X-axis. “-” on the X-axis corresponds to “no driver” controls. The Y-axis represents the time to reach 5 cm (seconds). Each bar represents the mean ± SE. Single or double asterisks indicate significant differences (p<0.05 or p<0.01, respectively). The numbers of flies tested in each experiment (between 7 and 20) are listed in S1 Table. For each of the disease models that expressed Htt-103Q or ATXN1-82Q we tested about 20 flies. Because the short Q controls showed stable performance we only about 8 flies for each group. The results of the statistical analyses are listed in S4 Table.

Next, to examine the motor activity of the fly models, we measured their climbing speed on the wall of a plastic cylinder. The cha>Htt-103Q and repo>Htt-103Q flies exhibited remarkably slow climbing at 7 days and died before 14 days (Fig. 3A, B, S1 and S4 Tables). The climbing abilities of the OK6>ATXN1-82Q and D42>ATXN1-82Q flies were also impaired at 7 days and 14 days (Fig. 3C, D, S1 and S4 Tables) and in OK6>Htt-103Q and ple>Htt-103Q at 14 days (Fig. 3B, S1 and S4 Tables). The climbing ability of cha>ATXN1-82Q flies was impaired at 7 days but not at 14 days (Fig. 3C, D, S1 and S4 Tables). Climbing speeds were decreased in D42>Htt-103Q and cha>ATXN1-82Q at 14 days and in ple>Htt-103Q at 7 days, although the difference was not statistically significant.

The pan-neuronal drivers (elav and 1407) showed no effect in the HD and SCA1 models. This might be due to the relatively low expression levels of these drivers (S2A Fig.). The most commonly used and originally described elav-Gal4 is *C155*, which is linked to the X chromosome. Previous reports that employed elav*^C155^* to express mutant Htt reported a locomotor phenotype [37]. Our elav-Gal4 is not *C155*, but a minor type linked to the 2^nd^ chromosome (P{GAL4-elav.L}2). It did not show homogenous pan-neuronal expression (S1 Fig.), which may explain why the effect was different from the previous reports.

### Spontaneous activity of the fly models

**Figure 4:**
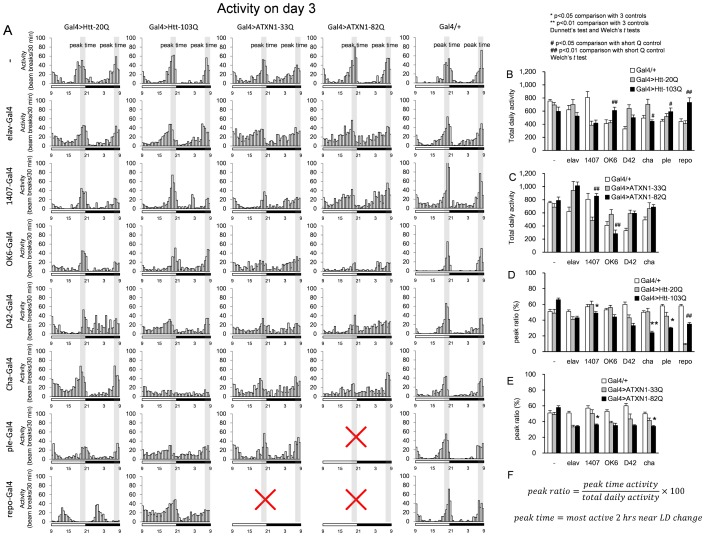
The daily activity was recorded for multiple fly models (A). Fly activities (number of beam breaks) during Zeitgeber rhythm were recorded on the 3^rd^ day and the ratios of the total beam break times are plotted. The genotypes are indicated on the left side and on the top of each graph. The LD cycle is indicated under each graph using open (light) and filled (dark) bars; the time is described below the bar. The peak times (7:00–9:00 and 19:00–21:00) are also indicated in each graph. Quantitative comparisons of total daily activity (B, C) or peak ratio (D, E) among three groups (Gal4/+, Gal4>normal polyQ, Gal4>mutant polyQ) are shown for Htt (B, D) and ATXN1 (C, E). Each bar represents the mean ± SE. Single and double asterisks indicate significant recovery compared to the three controls (Gal4, Gal4>normal polyQ, promoter negative-Gal4>mutant polyQ) (p<0.05 and p<0.01, respectively). # and ## indicate values significantly different from the short Q control (p<0.05 and p<0.01, respectively). The formula to calculate the peak ratio (F). The numbers of flies tested are listed in S1 Table. The results of the statistical analyses are listed in S5 Table.

We examined the circadian rhythm in the spontaneous activity of the model flies. First, the total daily activity was recorded under Zeitgeber time (LD 12/12 hours), and the histogram shows activities during each 30-minute window. In the control flies, two peaks of spontaneous activity were observed. One occurred before the light turned on (7:00–9:00), and the other occurred before the light turned off (19:00–21:00) (Fig. 4A). We extracted two parameters from the raw data for quantitative analysis: total daily activity and peak ratio (Fig. 4A, Fig. 4B–E). Total daily activity is the simple summation of activities from each 30-minute window. The calculation formula for peak ratio is shown in Fig. 4F. It reflects the maintenance of peak activity but not the accuracy of peak time. Therefore, the shift of peak time was judged only by qualitative evaluation.

The total daily activity values were paradoxically increased in OK6>Htt-103Q, ple>Htt-103Q, and repo>Htt-103Q flies (Fig. 4B). The results seem contradictory to the motor activity results (Fig. 3). However, while spontaneous activity was examined at 3 days after eclosion, the decline in motor activity was observed at 7 days after eclosion (Fig. 3). Total daily activity was decreased in cha>Htt-103Q flies (Fig. 4B) and in OK6>ATXN1-82Q flies (Fig. 4C).

Peak ratio was decreased in 1407>Htt103Q, cha>Htt103Q, ple>Htt103Q, 1407>ATXN1-82Q, and cha>ATXN1-82Q (Fig. 4D, E). In cha>polyQ flies, since total activity was also remarkably decreased, the activity peaks were nearly absent (Fig. 4A, S1 and S5 Tables). None of the models showed an increased peak ratio. Surprisingly, the peak time was greatly shifted from that of the repo>Htt-20Q (Fig. 4A) but not the repo>Htt-103Q flies (Fig. 4A), suggesting that normal Htt in glial cells contributes to the regulation of circadian rhythm.

### Morphologies of cha- and ple-positive neurons

**Figure 5:**
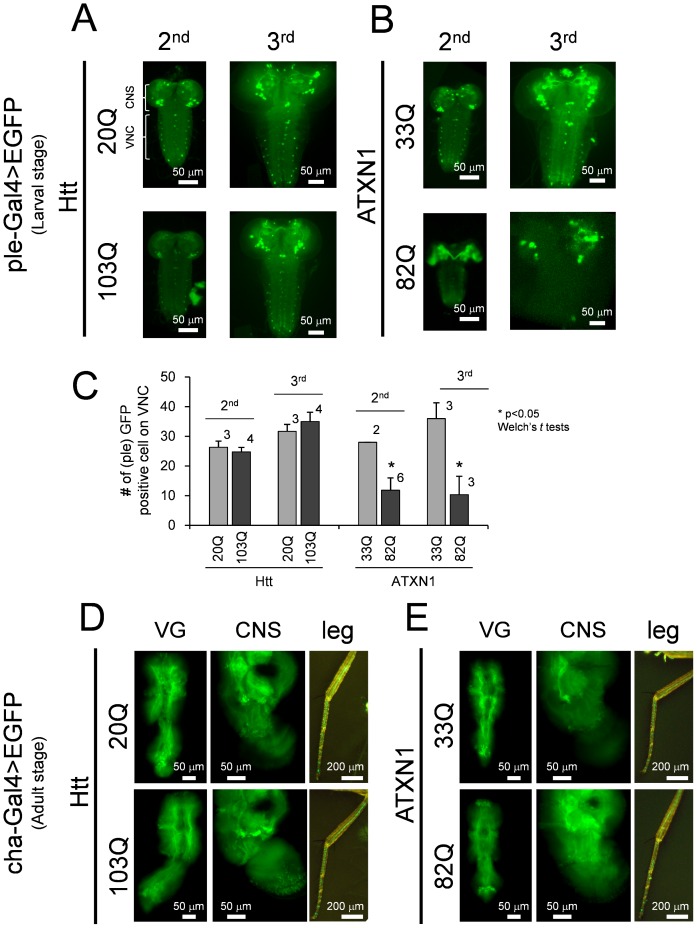
The EGFP expression patterns of cha- and ple-positive neurons in the SCA1 and HD models. The patterns of ple-Gal4 driven co-expression of EGFP and Htt (A) or ATXN1 (B). The left panels correspond to the 2^nd^ instar larvae, and the right panels correspond to the 3^rd^ instar larvae. CNS and VNC indicate the structure of the “central nervous system” and the “ventral nerve cord,” respectively. (C) Quantification of the number of EGFP signals in the ventral nerve cord in ple>polyQ larvae. The Y-axis represents the number of GFP positive cell bodies visible on ventral nerve cord. The X-axis represents the genotypes. The larval stages tested are depicted on each bar. Each bar represents the mean ± SE. The numbers of larva tested are presented on the shoulder of each bar. Single asterisks denote a significant difference between each short Q and long Q. The patterns of cha-Gal4 driven co-expression of EGFP and Htt (D) or ATXN1 (E). The left panels correspond to the “ventral ganglion,” the center panels correspond to the “central nervous system (brain),” and the right panels correspond to the peripheral neurons in a “leg.”

The phenotypes of the ple- and cha-driven HD and SCA1 models were very severe. Therefore, we analyzed the morphology of the dopaminergic (DA) and cholinergic (cha) neurons. We constructed transgenic fly models in which the polyQ protein and EGFP were expressed under the control of the same driver and observed the gross morphology (Fig. 5A, B). Ple>ATXN1-82Q was mostly lethal at the 2^nd^ instar, and only few larvae survived to the 3^rd^ instar. Normal brains structures (CNS) were rarely seen at the 2^nd^ stage (Fig. 5B). EGFP signals in DA neurons were decreased at the 2^nd^ stage in the VNC and at the 3^rd^ stage in the CNS of ple>ATXN1-82Q larvae (Fig. 5B). In contrast, the signal in DA neurons was not reduced in the ple>Htt-103Q larvae (Fig. 5A). The number of GFP-positive cells in the VNC was also decreased in ple>ATXN1-82Q but not in ple>Htt-103Q larvae (Fig. 5C). Since cha>ATXN1-82Q and cha>Htt-103Q flies survive to adulthood, we also examined the adult flies of these strains but could not detect any obvious change in the morphology of cha neurons (Fig. 5D, E). The GFP patterns in the VG, the CNS, and the leg peripheral neurons were quite similar.

### Effect of LiCl on viability and lifespans of the fly models

**Figure 6:**
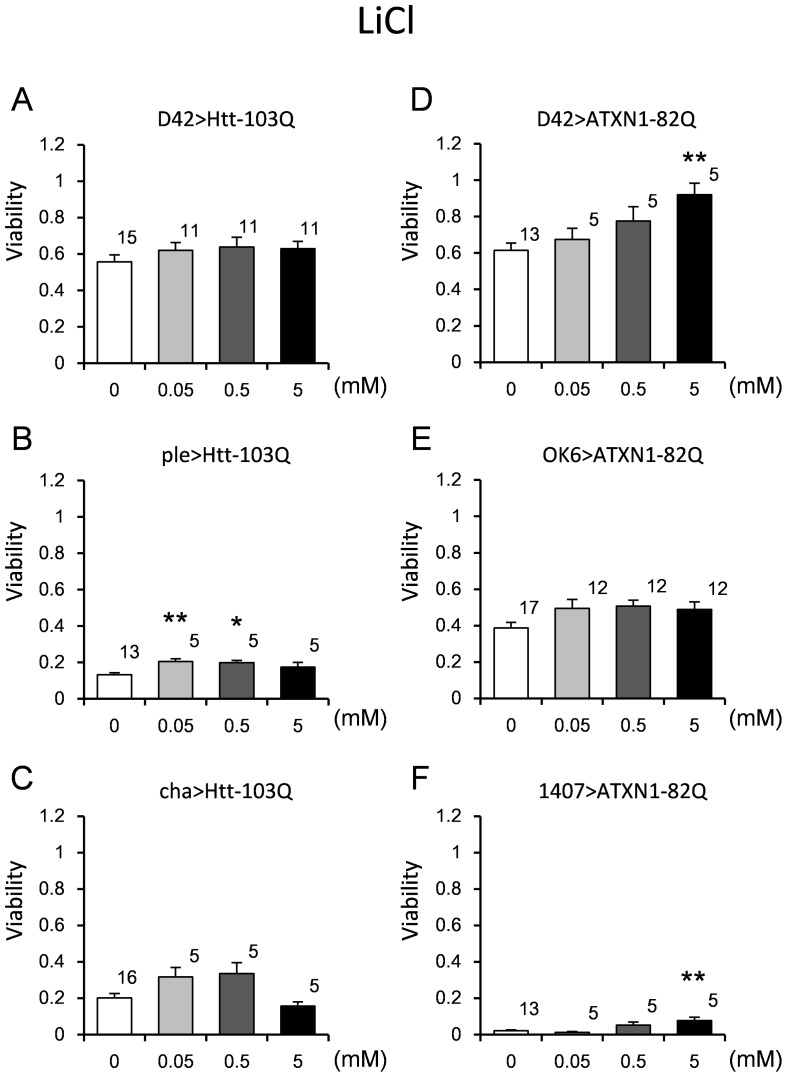
The effect of LiCl on the viability of the HD and SCA1 models. The viability was calculated as described in the “Materials and Methods” section. The concentrations are indicated on the X-axis, and the viability is indicated on the Y-axis. Three HD models (A–C) and 3 SCA1 models (D–F) were employed. The genotypes are described within each graph. Each bar represents the mean ± SE. The number of vials tested is indicated on the shoulder of each bar. Single and double asterisks denote significant differences (p<0.05 and 0.01, respectively). The results of the statistical analyses are listed in S6 Table.

We tested whether these models would be useful for quantitative evaluation of the effectiveness of drugs or modifier genes. Because the therapeutic effect of LiCl on an HD model has already been reported [11], we tested LiCl using our system. In this experiment, we examined the viability (see materials and methods) instead of the eclosion rate to promote efficient screening. We chose 3 fly models of each disease that exhibited a significant reduction in the eclosion rate (Fig. 1): D42>Htt-103Q, ple>Htt-103Q, cha>Htt-103Q, D42>ATXN1-82Q, OK6>ATXN1-82Q, and 1407>ATXN1-82Q. Supplementation of 0.05 mM or 0.5 mM LiCl throughout development significantly rescued the viability of ple>Htt-103Q (Fig. 6B, S6 Table). Interestingly, therapeutic effects in the SCA1 models (D42>ATXN1-82Q and 1407>ATXN1-82Q) were observed at a higher concentration (5 mM) (Fig. 6D, F, S6 Table). These findings indicate different mechanisms of rescue in the HD and SCA1 models. We confirmed that LiCl did not change the expression levels of the polyQ transgene (S2B Fig.).

**Figure 7:**
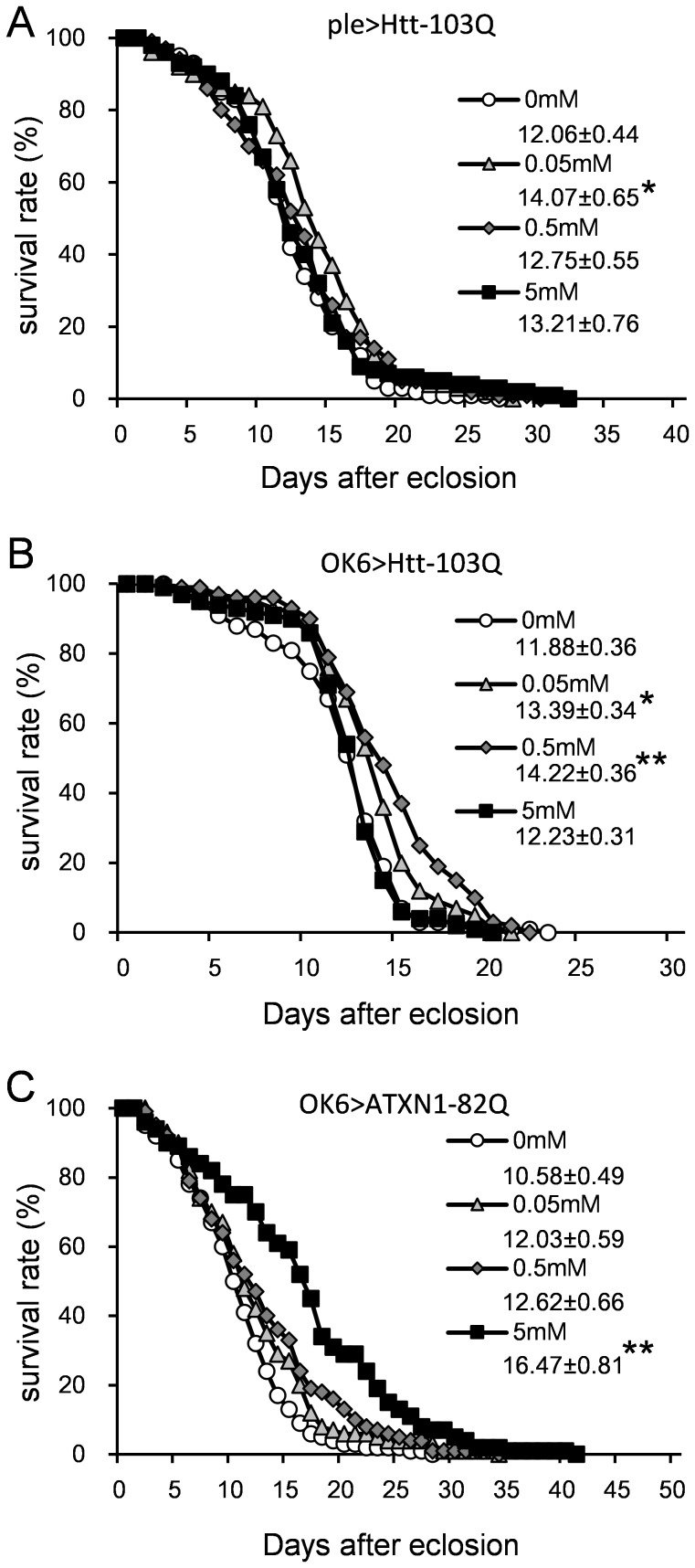
The effect of LiCl on the longevity of the HD and SCA1 models. The X-axis represents days, and the Y-axis represents the percent survival. All lines in a graph represent the same genotype. (A) ple>Htt-103Q. (B) OK6>Htt-103Q. (C) OK6>ATXN1-82Q. The mean lifespans (days) ± SE are shown below each symbol legend, with n = 100 for each line. Single or double asterisks indicate significant differences (p<0.05 or p<0.01, respectively). The results of the statistical analyses are listed in S7 Table.

We next evaluated the effect of LiCl on lifespan in models that showed remarkable lifespan shortening (Fig. 2): ple>Htt-103Q, OK6>Htt-103Q, and OK6>ATXN1-82Q. Supplementation with 0.05–5 mM LiCl significantly rescued the lifespan of the ple>Htt-103Q model; however, the effect was weak (Fig. 7A). A significant increase was observed in the OK6>Htt-103Q flies at 0.05 mM and 0.5 mM LiCl (Fig. 7B, S7 Table) and with the OK6>ATXN1-82Q flies at 5 mM (Fig. 7C, S7 Table). The effect of LiCl on the lifespans of OK6>Htt-103Q and OK6>ATXN1-82Q flies increased in a dose-dependent manner (Fig. 7B, C). The differences in the effective doses for the HD and SCA1 models were reproducibly confirmed. The recovery was considered specific for the disease models because LiCl did not rescue a short Q control (OK6>ATXN1-33Q, S4 Fig., S7 Table).

### Effect of butyrate on viability and lifespan of the fly models

**Figure 8:**
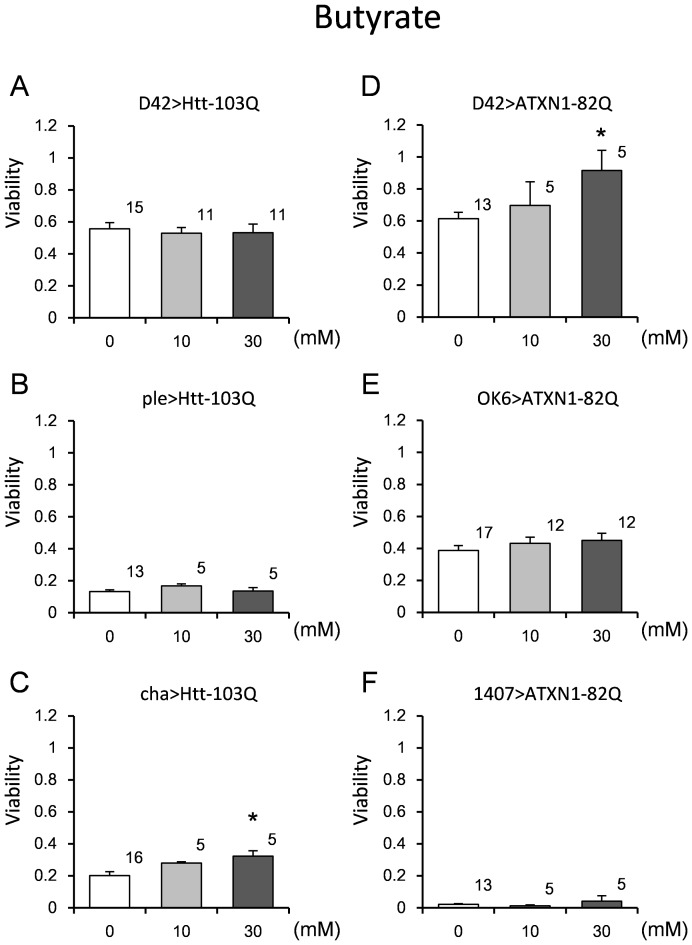
The effect of butyrate on the viability of the HD and SCA1 models. The viability was calculated as described in the “Materials and Methods” section. The concentrations are shown on the X-axis, and the viability is presented on the Y-axis. Three HD models (A–C) and 3 SCA1 models (D–F) were employed. The genotypes are described within each graph. For each bar, n = 5 was considered appropriate. Each bar represents the mean ± SE. The number of vials tested is indicated on the shoulder of each bar. Single asterisks indicate bars with significantly recovered values compared to the corresponding open bar (p<0.05). The results of the statistical analyses are listed in S6 Table.

We also tested the therapeutic effect of butyrate, since butyrate was reported to have a therapeutic effect on eye degeneration in an HD fly model [9]. We similarly employed the ple>Htt-103Q, OK6>Htt-103Q, and OK6>ATXN1-82Q models to examine the effect of butyrate. Butyrate rescued the viability of the D42>ATXN1-82Q and cha>Htt-103Q models at 30 mM (Fig. 8C, D, S6 Table). The D42>ATXN1-82Q model might be useful for chemical and genetic screens during development, since it responded to both chemicals (butyrate and LiCl). Therefore, we tried a combination therapy. We observed a slight increase in viability (S3B Fig.). We also confirmed that the chemical treatment did not change the expression level of the polyQ transgene (S2B Fig.).

**Figure 9:**
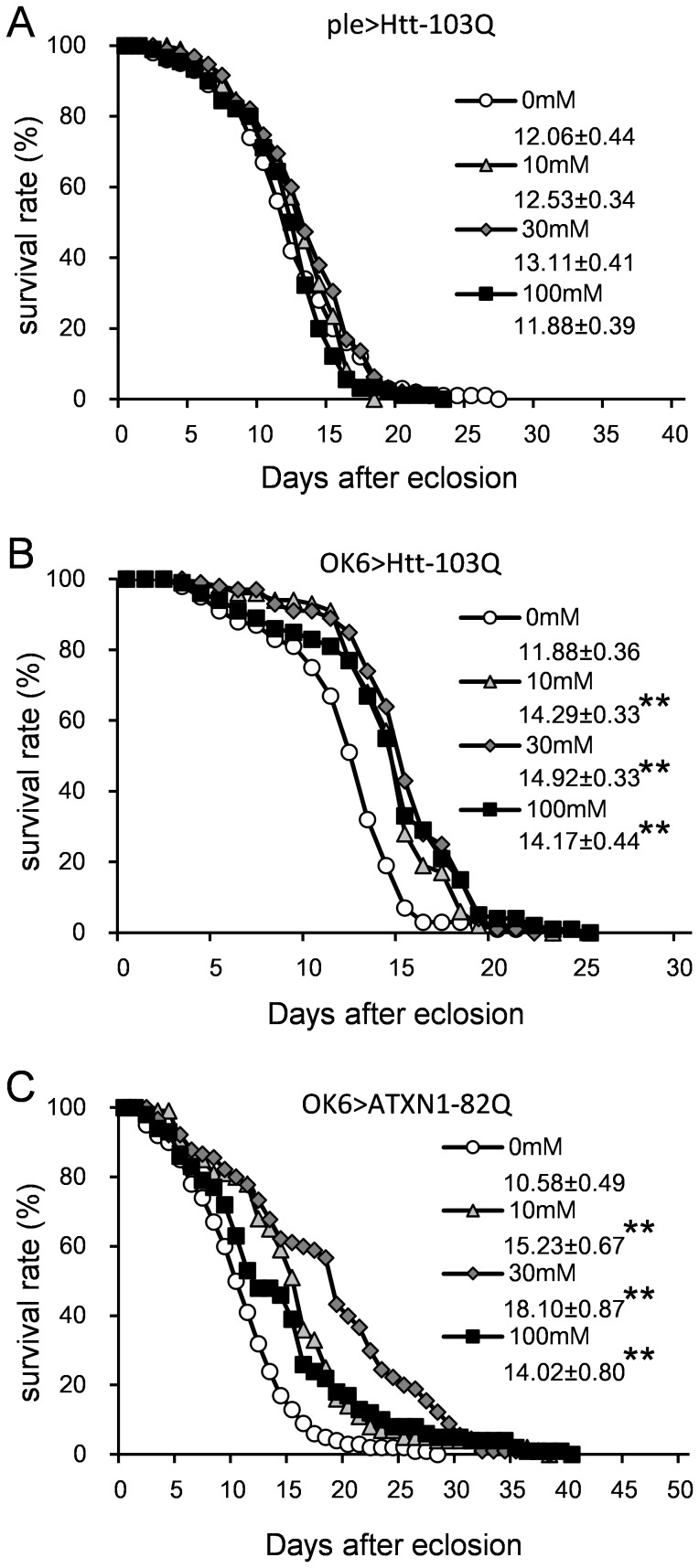
The effect of butyrate on the longevity of the HD and SCA1 models. The X-axis represents the number of days, and the Y-axis represents the survival percentage. All lines in the graph represent the same genotype. (A) ple>Htt-103Q. (B) OK6>Htt-103Q. (C) OK6>ATXN1-82Q. The mean lifespans (days) ± SE are shown below each symbol legend. N = 90 for OK6>ATXN1-82Q at 30 mM, and n = 100 for each other line. Double asterisks indicate significant differences (p<0.01). The results of the statistical analyses are listed in S7 Table.

We further evaluated the effect of butyrate on lifespan using the same method as for LiCl. A significant increase was observed in the lifespan of the OK6>Htt-103Q and OK6>ATXN1-82Q flies at all concentrations (10 mM, 30 mM and 100 mM) (Fig. 9B, C, S7 Table). However, such an increase was not observed in the ple>Htt-103Q flies (Fig. 9A). Notably, the increase due to butyrate was largest at 30 mM. Again, the effect was considered specific since butyrate did not rescue a short Q control (OK6>ATXN1-33Q, S4 Fig., S7 Table).

### Effect of JNK inhibitor on the viability of the fly models

**Figure 10:**
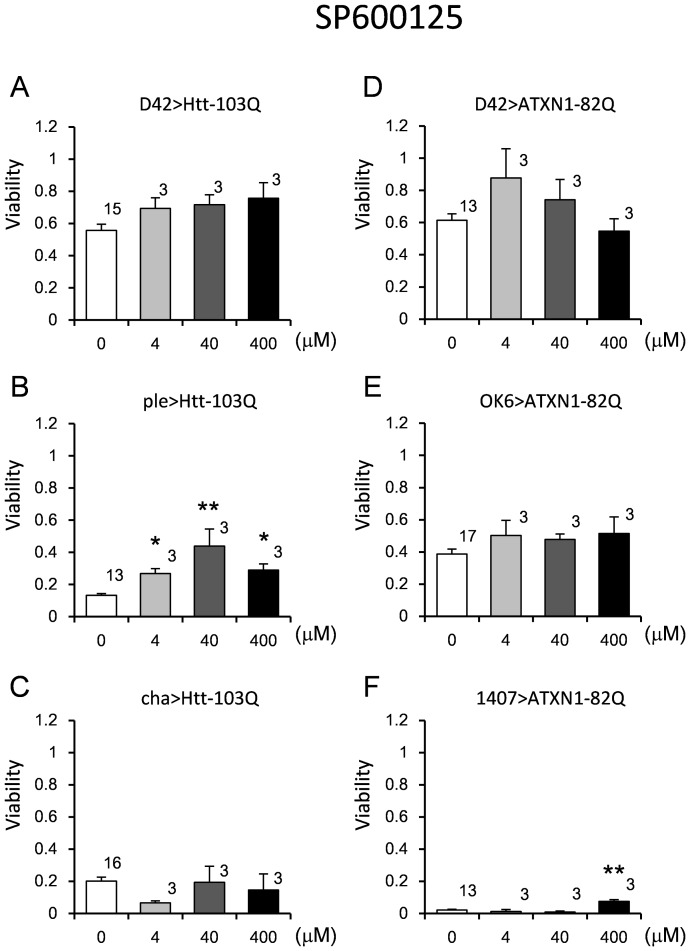
The effect of SP600125 on the viability of the HD and SCA1 models. The viability was calculated as described in the “Materials and Methods” section. The concentrations are presented on the X-axis, and the viability is presented on the Y-axis. Three HD models (A–C) and 3 SCA1 models (D–F) were employed. The genotypes are described within each graph. Each bar represents the mean ± SE. The number of vials tested is indicated on the shoulder of each bar. Single and double asterisks indicate significant differences (p<0.05 and 0.01, respectively). The results of the statistical analyses are listed in S6 Table.

We tested the therapeutic effect of a c-Jun N-terminal kinase (JNK) inhibitor, SP600125, on our fly models (Fig. 10). The neuroprotective effects of this inhibitor have been demonstrated *in vivo* and *in vitro* [38], [39]. The effect of SP600125 was assessed using the same method and fly strains used to evaluate the effect of LiCl. We observed significant rescue of lifespan in the ple>Htt-103Q and 1407>ATXN1-82Q models. The therapeutic effect of SP600125 was observed at a wide range of concentrations in ple>Htt-103Q (Fig. 10B, S6 Table), while only a weak recovery was observed using the highest concentration in 1407>ATXN1-82Q (Fig. 10F, S6 Table). We confirmed that the JNK inhibitor did not change the expression levels of the polyQ transgene (S2B Fig.).

## Discussion

### Different stage-specific toxicities of Htt and ATXN1

ATXN1-82Q induced severe developmental phenotypes, especially when expressed by the ple and repo drivers. In addition to its expression in dopaminergic neurons, ple (DTH) is expressed in the hypoderm where it is important for cuticle hardening and embryonic viability [40], [41]. Thus, expression of ATXN1-82Q in the hypoderm may contribute to the severe embryonic lethality seen in ple>ATXN1-82Q.

We previously tested the effect of glial expression of mutant Htt and ATXN1 using the gcm and repo drivers [21]. Both drivers induce glial cell lineage expression, but their expression patterns are different. Gcm is expressed in neuroglioblasts, and its expression is reduced in immature glia. On the other hand, repo expression begins in glioblasts and its expression stays high in immature and mature glia. ATXN1-82Q expression by gcm generates a more severe phenotype, suggesting that the developmental effect of ATXN1-82Q on the earliest stages of glial development is more prominent than that at later stages.

The developmental effect of Htt-103Q is generally weak, except when its expression is induced by cha. Progressive effects after eclosion by cha-induced expression were more severe in the Htt-103Q versus the ATXN1-82Q model. This exception might be explained by cell type-specific vulnerability in cholinergic neurons.

It is important to note that each driver induced similar (but not equal) expression of both Htt-103Q and ATXN1-82Q mRNA (S2A Fig.). Additionally, because of the low expression levels and restricted expression patterns we were unable to estimate protein levels by western blotting. Since each polyQ protein (Htt-20Q, 103Q and ATXN1-33Q, 82Q) might have its own degradation rate, the mRNA levels might not reflect protein levels and the protein levels may vary. Thus, the long Q proteins in our models could be present at relatively high levels because of their slow degradation rate [19]. While such slow degradation of pathogenic proteins is believed to be a part of pathogenesis, we cannot exclude the possibility that the different phenotypes of the HD and SCA1 models might be due to different protein levels.

### Utility of fly models for screening

This study provided multiple fly polyQ disease models and information about their behaviors. In addition, we tested the therapeutic effects of drugs that had previously been shown to be effective (Figs. 6–10). Our positive results provide proof-of-principle for the use of these fly models in more large-scale therapy screening experiments. Importantly, since the phenotypes were variable, the appropriate model can be selected depending on the purpose of drug development.

Despite the advantages of this system, several caveats remain. First, the efficiency and sensitivity of this drug screening method should be carefully considered. For instance, a lifespan test requires 20–30 days. Therefore, serial starting time points would be required to increase the number of drugs that can be screened. In addition, since large amounts of each drug are required for this method, it may not be appropriate when the quantities of each drug in a chemical library are limited.

The second set of caveats involves the disease specific vulnerabilities for distinct neuronal subtypes and cell types. Cholinergic neurons were vulnerable to mutant Htt but not to mutant ATXN1. Dopaminergic neurons seemed to be vulnerable to both mutant Htt and ATXN1, but were somewhat more sensitive to the latter. Incidentally, dopaminergic neurons seemed to be more vulnerable than motor neurons to both mutant Htt and ATXN1 (Table 1). The higher expression level of mutant Htt in dopaminergic neurons could be related to the strong phenotypes (S2A Fig.). However, similar expression levels of mutant Htt and ATXN1 in cholinergic and motor neurons suggest that the severity of phenotypes is not related to expression differences in these models (S2A Fig., Table 2). Vulnerability to specific neuronal types for each disease should be carefully considered in further studies. Neuronal and cell type specificity should be considered when selecting the model for drug screening. Moreover, there are limitations to equating Drosophila neurons with human neurons that express the same neurotransmitter. For example, motor neurons are cholinergic in humans but glutamatergic in *Drosophila*. Such differences should be considered when using *Drosophila* as a convenient model for drug screening and validation.

Finally, disease genes may affect both development and adult homeostasis. ATXN1 can cause developmental as well as late-onset phenotypes in Drosophila. The developmental effects of ATXN1 were also reported previously in mice [42]. This consistency across species might suggest that SCA1 can cause developmental pathologies in human patients. Since drugs that are effective at developmental stages might be different from those that are effective for progressive symptoms in adulthood, one should consider the intended target stage of a drug for clinical application.

## Conclusion

The systematic analysis of Drosophila polyQ models revealed multiple phenotypes reflecting cell type-specific, neuronal subtype-specific, developmental stage-specific, and disease gene-specific dysfunctions. Our fly model system would be useful to screen drugs in therapeutics development, although some issues remain to be resolved to improve efficiency.

## Supporting Information

S1 Fig **The patterns and levels of EGFP expression for each driver.** The central nervous system (CNS, A–G) and ventral ganglion (VG, H–N) were observed under two different conditions (adjusted gain and expanded gain). First, in order to compare expression levels between drivers we obtained all images by using the same conditions (laser power, gain, offset) (adjusted gain). The conditions were determined based on the weakest driver (ple-Gal4). Second, in order to observe the detailed morphology we set up separate conditions for each driver based on sample brightness to expand the dynamic range and avoid saturation (expanded gain).
(TIF)

S2 Fig (A) The results of real-time PCR performed using an absolute quantitation method. Comparison of expression levels in each of the models and controls. The X-axis represents the genotypes (driver and UAS combinations). The Y-axis represents the mRNA copy number. Each bar represents the mean ± SE, n = 3. (B) The expression levels of ATXN1-82Q and Htt-103Q in chemical-treated flies were determined by the absolute quantification method. Fly genotypes and chemicals are shown under and on the bars, respectively. The most effective concentrations of chemicals were chosen for the quantification (5 mM LiCl and 400 µM SP600125 for 1407>ATXN1-82Q; 30 mM Butyrate for cha>Htt-103Q; 0.05 mM LiCl and 40 µM SP600125 for ple>Htt-103Q; and 5 mM LiCl, 30 mM Butyrate, and the combination for D42>ATXN1-82Q). The Y-axis indicates relative expression levels of polyQ mRNA in chemical-treated flies in comparison to that in control flies (no chemical). Each bar represents mean ± SE (n = 3).
(TIF)

S3 Fig (A) The viability of each driver and marker were estimated. The labels on the X-axis are the genotypes of the male flies used. The males are the same flies as those used for the viability test in the chemical screening. Driver/marker hetero males were crossed with WT virgin females. The numbers of F1 flies with the driver or marker were counted. The driver/marker ratio is shown on each bar. The ratio should be 1 (indicated by the dotted line) when the viabilities of the driver and the marker are the same. The values were employed to compensate for the results of the viability tests (Figs. 6, 8 and 10). Each bar represents the mean ± SE, n = 5. (B) Effect of combination therapy of LiCl and butyrate (BA) on D42>ATXN1-82Q flies. Each bar represents the mean ± SE. The number of vials tested is indicated on the shoulder of each bar. Single and double asterisks indicate significant differences (p<0.05 and 0.01, respectively).
(TIF)

S4 Fig **The effect of LiCl and butyrate (BA) on the longevity of a short Q control.** The X-axis represents the days, and the Y-axis represents the survival percentage. All lines in the graph represent OK6>ATXN1-33Q. Lines are explained in the inset. The mean lifespan (days) ± SE is below each symbol legend, with n = 45 for each line. There was no significant difference between the control and each chemical treatment. The results of the statistical analyses are listed in S7 Table.
(TIF)

S1 Table **Summary of the number of tests (** **Figs. 1** **–** **4** **).** N represents the number of vials for the eclosion test and the number of flies for the other assays.
(DOCX)

S2 Table **Results of the statistical analyses for the eclosion rate (** **Fig. 1** **).**
(DOCX)

S3 Table **Results of the statistical analyses for longevity (** **Fig. 2** **).**
(DOCX)

S4 Table **Results of the statistical analyses of the negative geotaxis assay (** **Fig. 3** **).**
(DOCX)

S5 Table **Results of the statistical analyses of the spontaneous activity assay (** **Fig. 4** **).**
(DOCX)

S6 Table **Results of the statistical analyses for viability (** **Figs. 6** **,** **8** **,** **10** **).**
(DOCX)

S7 Table **Results of the statistical analyses of the effects of LiCl and butyrate on lifespan (** **Figs. 7** **and** **9** **,** **S4 Fig.** **).**
(DOCX)
