# Supplementary figures and images for: Systematic Analysis of Fly Models with Multiple Drivers Reveals Different Effects of Ataxin-1 and Huntingtin in Neuron Subtype-Specific Expression
Source: PLoS One. 2014 Dec 31;9(12):e116567. doi: 10.1371/journal.pone.0116567 (PMC4281079; doi:10.1371/journal.pone.0116567)

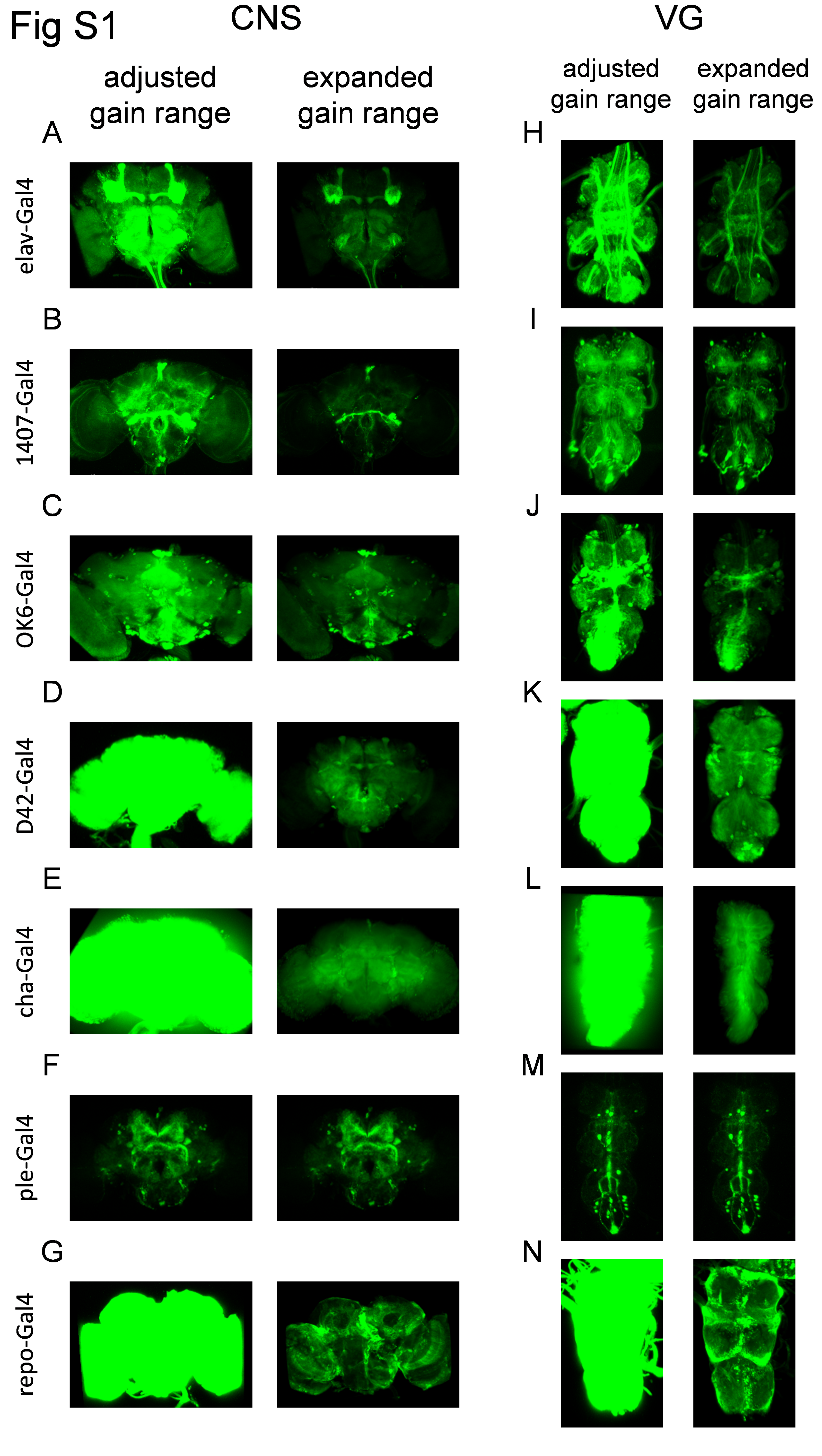

Supplement: S1 Fig — The patterns and levels of EGFP expression for each driver. The central nervous system (CNS, A–G) and ventral ganglion (VG, H–N) were observed under two different conditions (adjusted gain and expanded gain). First, in order to compare expression levels between drivers we obtained all images by using the same conditions (laser power, gain, offset) (adjusted gain). The conditions were determined based on the weakest driver (ple-Gal4). Second, in order to observe the detailed morphology we set up separate conditions for each driver based on sample brightness to expand the dynamic range and avoid saturation (expanded gain). (TIF) [file pone.0116567.s001.tif]

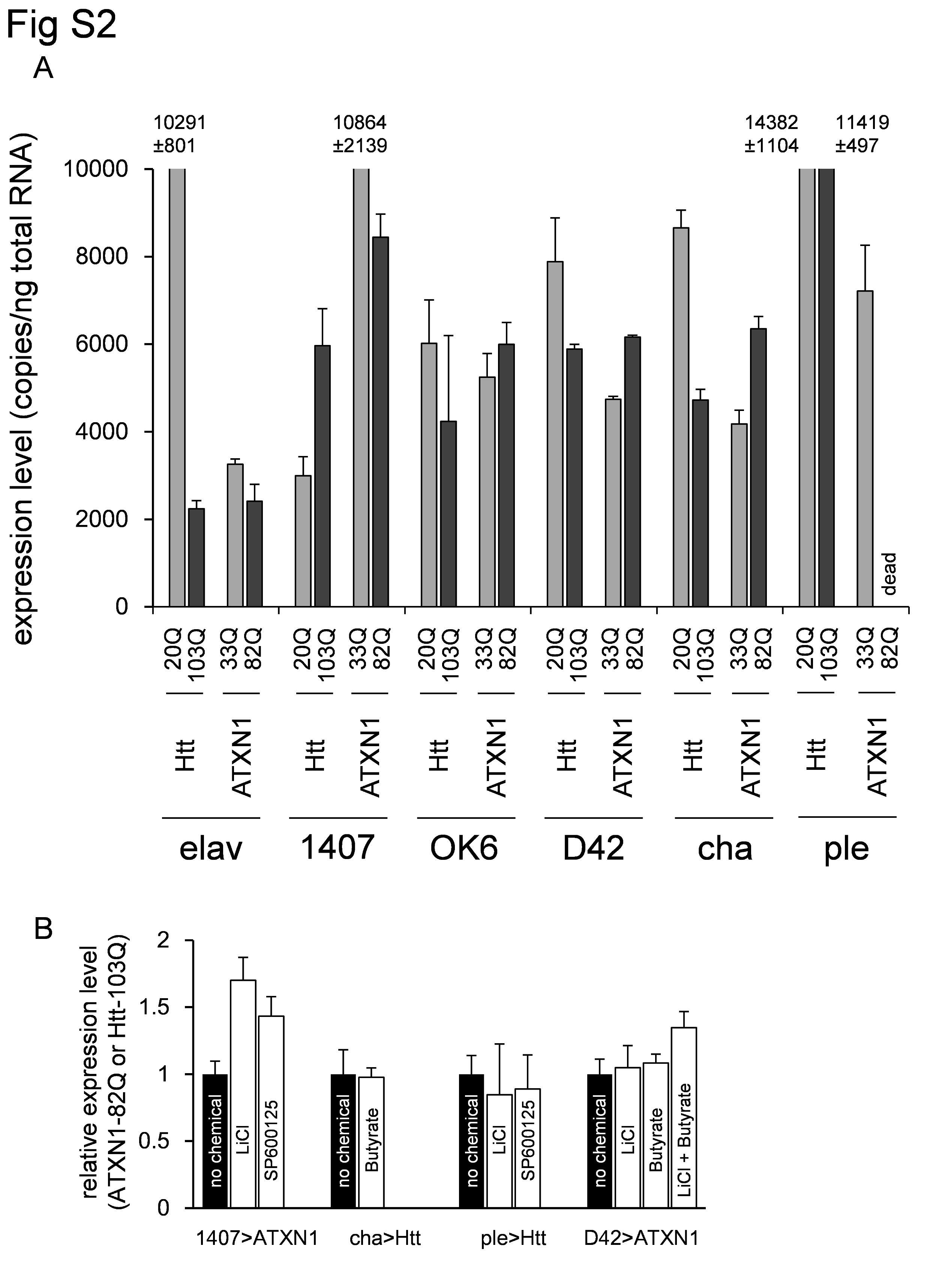

Supplement: S2 Fig — (A) The results of real-time PCR performed using an absolute quantitation method. Comparison of expression levels in each of the models and controls. The X-axis represents the genotypes (driver and UAS combinations). The Y-axis represents the mRNA copy number. Each bar represents the mean ± SE, n = 3. (B) The expression levels of ATXN1-82Q and Htt-103Q in chemical-treated flies were determined by the absolute quantification method. Fly genotypes and chemicals are shown under and on the bars, respectively. The most effective concentrations of chemicals were chosen for the quantification (5 mM LiCl and 400 µM SP600125 for 1407>ATXN1-82Q; 30 mM Butyrate for cha>Htt-103Q; 0.05 mM LiCl and 40 µM SP600125 for ple>Htt-103Q; and 5 mM LiCl, 30 mM Butyrate, and the combination for D42>ATXN1-82Q). The Y-axis indicates relative expression levels of polyQ mRNA in chemical-treated flies in comparison to that in control flies (no chemical). Each bar represents mean ± SE (n = 3). (TIF) [file pone.0116567.s002.tif]

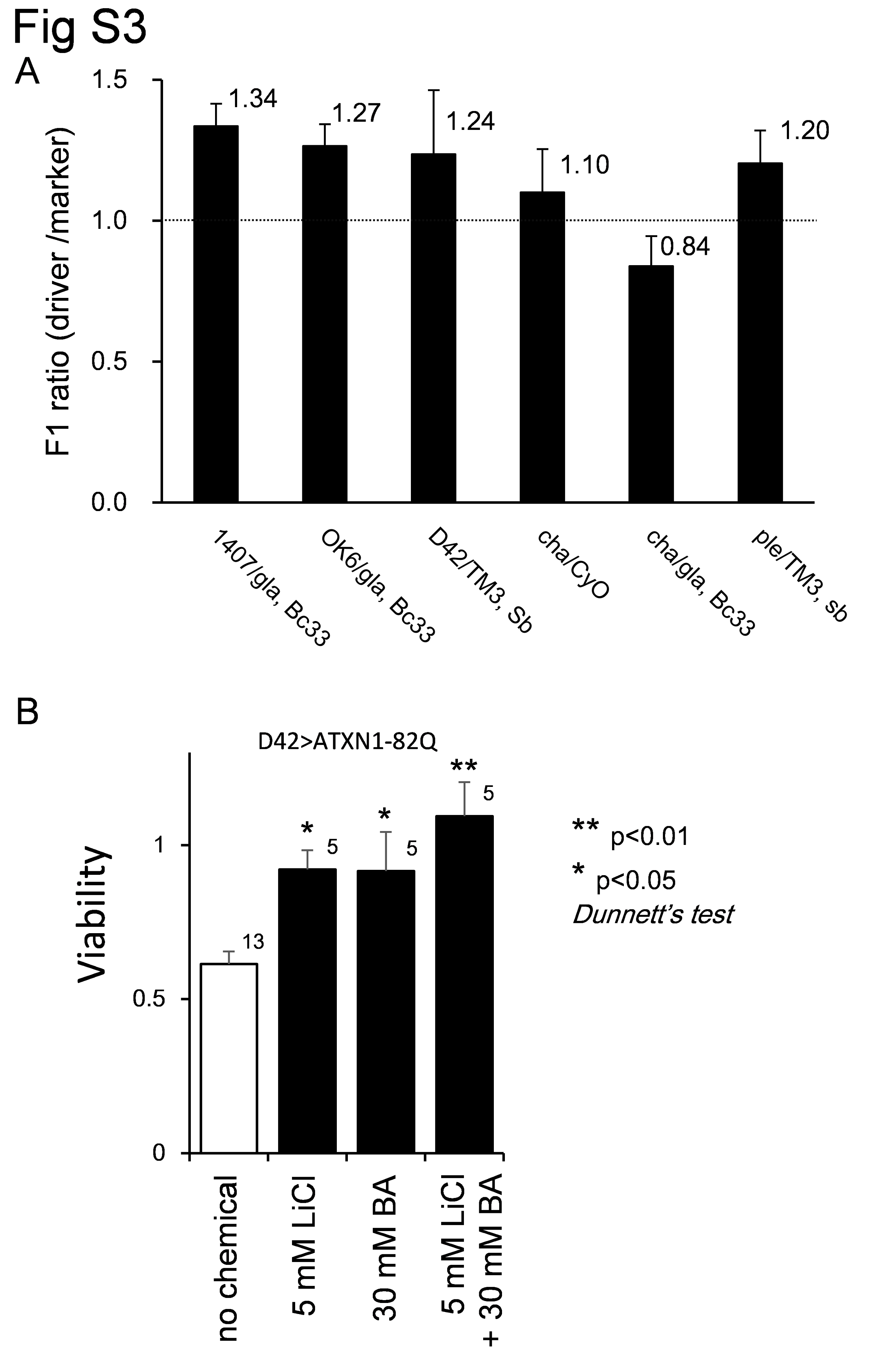

Supplement: S3 Fig — (A) The viability of each driver and marker were estimated. The labels on the X-axis are the genotypes of the male flies used. The males are the same flies as those used for the viability test in the chemical screening. Driver/marker hetero males were crossed with WT virgin females. The numbers of F1 flies with the driver or marker were counted. The driver/marker ratio is shown on each bar. The ratio should be 1 (indicated by the dotted line) when the viabilities of the driver and the marker are the same. The values were employed to compensate for the results of the viability tests (Figs. 6, 8 and 10). Each bar represents the mean ± SE, n = 5. (B) Effect of combination therapy of LiCl and butyrate (BA) on D42>ATXN1-82Q flies. Each bar represents the mean ± SE. The number of vials tested is indicated on the shoulder of each bar. Single and double asterisks indicate significant differences (p<0.05 and 0.01, respectively). (TIF) [file pone.0116567.s003.tif]

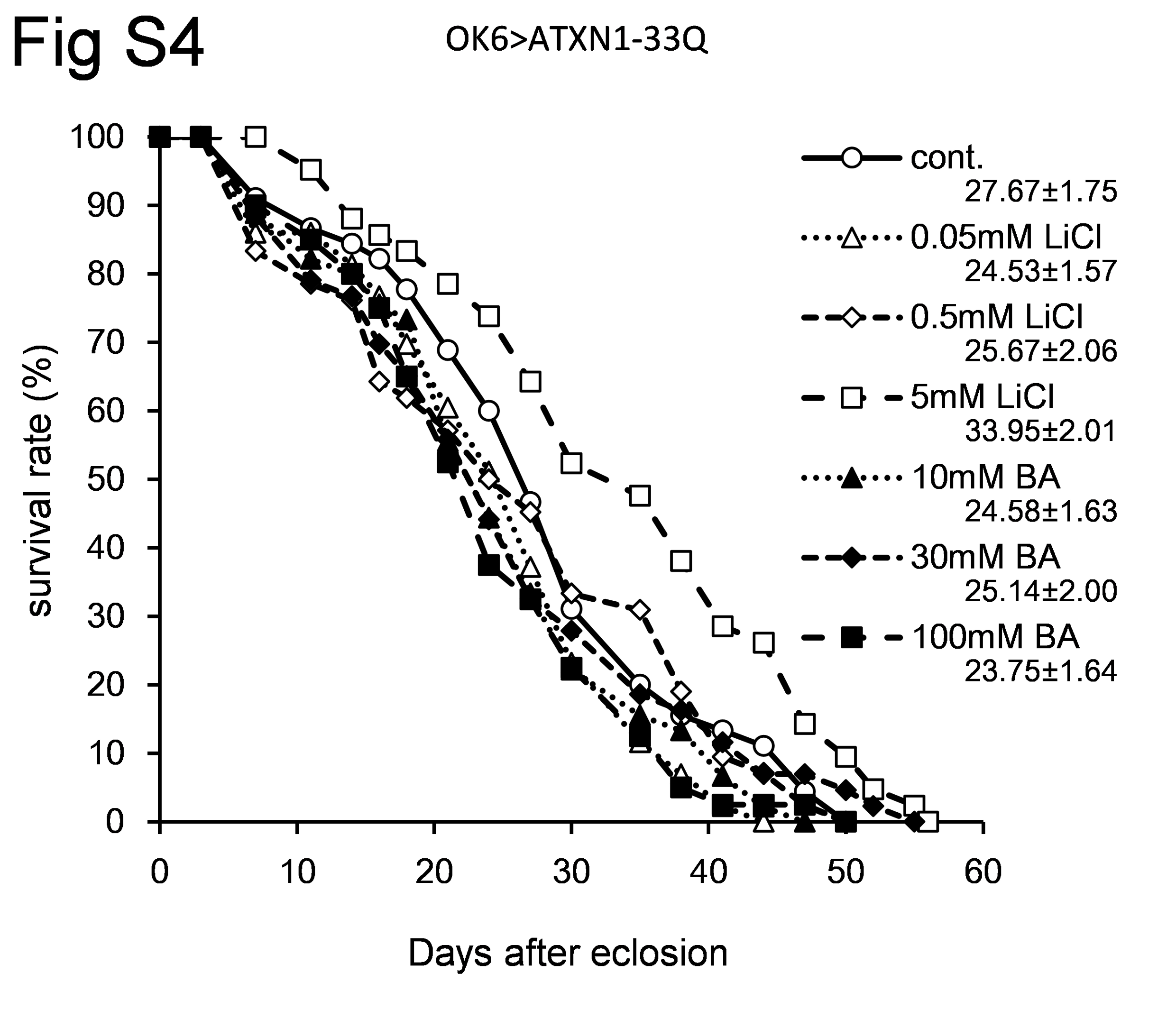

Supplement: S4 Fig — The effect of LiCl and butyrate (BA) on the longevity of a short Q control. The X-axis represents the days, and the Y-axis represents the survival percentage. All lines in the graph represent OK6>ATXN1-33Q. Lines are explained in the inset. The mean lifespan (days) ± SE is below each symbol legend, with n = 45 for each line. There was no significant difference between the control and each chemical treatment. The results of the statistical analyses are listed in S7 Table. (TIF) [file pone.0116567.s004.tif]
