# Supplementary material for: Systematic Analysis of Fly Models with Multiple Drivers Reveals Different Effects of Ataxin-1 and Huntingtin in Neuron Subtype-Specific Expression
Source: PLoS One. 2014 Dec 31;9(12):e116567. doi: 10.1371/journal.pone.0116567 (PMC4281079; doi:10.1371/journal.pone.0116567)
Supplement: S4 Table — Results of the statistical analyses of the negative geotaxis assay ( Fig. 3 ). (DOCX) [file pone.0116567.s008.docx]

Table S4.
